# Are co-attention effects on memory limited to group members? A registered conceptual replication and extension of Shteynberg (2010)

**DOI:** 10.1007/s00426-026-02329-y

**Published:** 2026-06-15

**Authors:** Shaheed Azaad, Dirk Scheele, Garriy Shteynberg, Gerald Echterhoff

**Affiliations:** 1 Department of Psychology, Social Psychology Group, University of Münster, Münster, Germany; 2 Department of Social Neuroscience, Faculty of Medicine, Ruhr University Bochum, Bochum, Germany; 3 Research Center One Health Ruhr of the University Alliance Ruhr, Ruhr University Bochum, Bochum, Germany; 4 University of Tennessee, Knoxville, TN USA

## Abstract

Co-attention has been shown to enhance memory. Although this effect has been replicated in different social contexts, its minimal conditions remain unclear. Features of previous experiments have been conducive to collaboration, such as participants completing complementary tasks or experiencing a minimal ingroup manipulation. Consequently, it remains unknown whether co-attention effects on memory reflect a fundamental motivation to represent the mental states of those around us, or whether they are limited to coordination contexts. The proposed studies will test whether mere passive co-attention with a stranger is sufficient to enhance memory. We plan two experiments to: (1) conceptually replicate Shteynberg’s (2010; JPSP) finding that seeing the same (versus different) stimuli alongside an in-group member enhances memory, and (2) test whether mere co-attention enhances memory, absent the group manipulation.

Our ability to coordinate with others relies on our forming a common understanding of our environment, task, and capacity for action (Azaad et al., 2021). One way in which we accomplish this is by engaging in shared, or co-, attention with those around us (Shteynberg, 2015; Tomasello, 1995). Even in infancy, we achieve co-attention by following others’ gaze (Senju & Csibra, 2008) or gesturing to others to follow ours (Southgate et al., 2007). In addition to influencing *what* information we process, co-attention seems to impact *how* we process information – a notable example of this is the finding that co-attended stimuli are more likely to be remembered (for reviews, see Echterhoff & Kopietz, 2017; Shteynberg, 2014).

Explanations of co-attention effects on memory tend to agree that they serve to enable us to coordinate with others (Elekes & Sebanz, 2020; Shteynberg, 2015). In their *social epistemic* account, Elekes and Sebanz (2020) propose that co-attention effects on memory reflect a fundamental motivation to represent others’ knowledge states. Shteynberg’s (2015) *social tuning* hypothesis adds that this motivation is especially strong for relationally close others. Extant studies suggest that co-attention effects on memory are more likely to emerge when: (1) participants assume their partners intentionally process simultaneously attended stimuli, and (2) feel that their partners form an ad-hoc ingroup. However, the minimal conditions for such effects remain unknown. The present paper seeks to test whether mere co-attention with a stranger can facilitate memory without experienced similarity or in-group membership.

## Co-attention effects on memory

One example of co-attention effects on memory comes from Shteynberg’s (2010) *social tuning effect*. Shteynberg (2010; Study 2) recruited groups of three participants to complete a word recognition task. Before exposure to these words, participants selected an avatar of one of five colours to represent them throughout the experiment. Shteynberg (2010) manipulated perceived group membership by telling half of his participants that their co-participants picked the same colour as them (ingroup condition) and the remaining that their co-participants picked different colours (non-group condition). After passively viewing a word list together with two other participants seated in different rooms, participants completed a recognition task that involved identifying the previously presented words from a second list that also contained new words. Participants in the ingroup condition displayed superior recognition performance than those in the non-group condition (see also He et al., 2011).

In a follow-up experiment, Shteynberg (2010; Study 3) sought to rule out the possibility that mere co-presence could explain the improvement in recognition performance. Participants in this experiment performed a change detection task in the same two (in-group/non-group) conditions as in his Study 2, with an additional condition in which participants completed the task with in-group members who ostensibly saw different stimuli. Participants in the in-group same-stimuli condition were more accurate than those in the in-group different-stimuli and non-group same-stimuli conditions. Shteynberg (2010) proposes that people ‘tune in’ – by allocating a larger proportion of cognitive resources - to stimuli assumed to be experienced with their social in-group. As a result, representations of co-experienced stimuli are rendered more *accessible*, or more readily activated.

Research on Eskenazi et al.’s (2013) *joint memory effect* suggests that co-attention can augment memory even when there is no information suggesting that the partners are from participants’ social in-group. Participants in Eskenazi et al. completed a word categorisation task in which they responded to words from one of three categories (e.g. animals) while ignoring words from the other two (e.g. fruits/vegetables and household items). In a subsequent memory test, participants were more likely to recall words from their target category. Strikingly, when participants performed the same task with a partner who responded to one of the remaining two categories, they also recalled more words from their partner’s category. Participants did not recall words from the unassigned stimulus category in the joint categorisation task with greater likelihood than in the solo version. This finding emerged despite participants nevertheless needing to attend to words from the unassigned category to decide whether to respond.

According to Elekes and Sebanz’s (2020) *social epistemic* account of joint memory effects, the co-attention effect on memory reflects humans’ fundamental motivation to represent and monitor others’ knowledge states (Echterhoff et al., 2009). In other words, shared attention enables us to form common ground with others by prioritising co-attended stimuli. Due to design features of co-attention studies, there remains an open question about the minimal conditions under which people experience this motivation. Specifically, it is unknown whether *mere* co-attention, without group manipulations, a shared task, or other preconditions, is sufficient to facilitate memory.

The conditions under which co-attention effects on memory occur may point to their underlying mechanisms. One possibility is that these effects exist to facilitate coordination with others. In this case, we would expect these effects to occur only when there is some cue indicating future joint action with a co-attendee. This would explain why, in joint memory tasks, memory is not enhanced for stimuli to which neither participant responds, despite them being viewed together (Eskenazi et al., 2013). Working on a complementary task alongside another might spontaneously elicit feelings of coordination (Sebanz et al., 2003), leading participants to dedicate greater cognitive resources to the task-relevant (i.e., responded-to) stimuli. Another possibility is that social cues to future coordination render stimuli relevant even in the absence of a collaborative task. For instance, people are especially likely to coordinate spontaneously when paired with an ingroup member (McClung et al., 2013). This might explain why mere co-attention with an ingroup member is sufficient to boost memory (Shteynberg, 2010).

Alternatively, as Elekes and Sebanz (2020) propose, people might be fundamentally motivated to represent others’ knowledge states. In this case, we might expect that mere co-attention – i.e., without coordination cues - could enhance memory for co-attended stimuli. While it is true that memory for unassigned stimuli is not enhanced in joint memory (Eskenazi et al., 2013) paradigms, this might be due to the task context. Because participants are instructed to respond to specific categories of stimuli while ignoring the others, they might have actively suppressed their processing of unassigned stimuli to improve task performance. Such active suppression could result in poorer memory for stimuli compared to passive-viewing contexts without assignment of relevant and irrelevant task categories. Indeed, studies on shared experiences of stimuli suggest that merely sharing an experience with another can amplify the experience (Boothby et al., 2014). This amplification might come from a greater dedication of cognitive resources to co-attended stimuli, which would consequently improve our memory for them (Challis et al., 1996).

### The proposed studies

The present studies seek to test whether co-attention enhances memory for passively viewed stimuli without a group manipulation. To this end, we plan two experiments based on Shteynberg’s (2010) social tuning paradigm. First, we will seek to conceptually replicate the effect of seeing the same versus different stimuli (i.e., co-attention) with similar others as in Shteynberg’s Study 3 (Experiment 1). Rather than using a change-detection paradigm as Shteynberg did in his study, we will test for memory effects in a word recognition task.

We plan for three possibilities based on Experiment 1’s results. (1) If we find a significant effect of co-attention, we will continue to Experiment 2, which will replicate Experiment 1 without a group induction to test whether mere co-attention enhances memory. (2) If we do not find a significant effect of co-attention in Experiment 1, *and* our equivalence test indicates there was no effect of co-attention, then we will not conduct a second experiment. This is because Experiment 1’s conditions should be at least, if not more, conducive to co-attention effects than those of Experiment 2 (Shteynberg, 2010). In other words, it is very unlikely that we would find an effect in Experiment 2, given that we did not in Experiment 1. Rather, we would consider our study a failed conceptual replication of Shteynberg’s Study 3. (3) If we do not find a significant effect in Experiment 1, but equivalence tests do *not* yield evidence for the null hypothesis either, it would suggest that there might have been an effect of co-attention that our study was too underpowered to detect. In this case, we will conduct Experiment 2 with a sample size determined by the effect size from Experiment 1.

#### Experiment 1

In Experiment 1, we will conceptually replicate Shteynberg’s (2010) Study 3. Participants will complete a word recognition task in parallel with a confederate. Critically, we will manipulate co-attention by telling participants that their partner will see either the same or different stimuli. In Shteynberg’s study, participants demonstrated better memory, as measured by their ability to detect a change in a previously seen painting, when they believed their co-participants had seen the same stimulus. We predict a similar effect: if social tuning effects rely on shared attention, we should see improved recognition performance when participants believe their partner will see the same (vs. different) stimuli.

Our experiment will differ from Shteynberg’s (2010) in two substantial ways. First, instead of recruiting groups of three, participants will perform the task with just one confederate. Since group membership effects on co-action (Aquino et al., 2015) and joint memory effects (Eskenazi et al., 2013) have both been shown to occur in dyads, it is unlikely that the presence of a third person is a necessary precondition for our focal effect. Second, participants will perform the task in the same room as the confederate. We made this change to maximise the impact of co-attention since work on the joint memory effect has shown that it decreases with distance between participants (Boothby et al., 2016; Wagner et al., 2017).

## Method

### Participants

Although Experiment 1 is conceptually similar to Shteynberg’s (2010) Study 3, we performed a power calculation based on his word recognition experiment test for the effect of group similarity. This is because (1) Shteynberg’s Study 3 reports an effect from a logistic regression with three conditions, which does not map onto our design, and (2) our study will use a word recognition task instead of a change detection one, and (3) the effects of group similarity and stimulus similarity in Shteynberg’s Study 3 were of equal magnitude.

While we computed an effect of *d* = 0.74 from Shteynberg’s (2010) reported data, we opted to base our power calculation on a moderate effect of *d* = 0.50 instead, to ensure adequate power in case the true effect is smaller. A power analysis using G*Power 3.1 (Faul et al., 2007) for a one-sided independent samples *t*-test revealed that to detect an effect of *d* = 0.50 with 0.90 power, we will need a sample of 140 participants − 70 in each between-subjects condition. Accordingly, we will recruit 140 participants, aged 18–65, with normal or corrected-to-normal vision. All experiments are approved by the University of Münster’s Psychology and Sports Science Ethics Committee (Ethikkommission des Fachbereichs 7).

### Procedure

We will invite participants to our lab, where they will be introduced to the experiment alongside a confederate. Participants will then continue to a computer task, with the confederate seated opposite them so that their screens are back-to-back.

After reading the task instructions, participants will see a screen on which they can choose one of five coloured avatars (red, green, blue, orange, or purple) to represent them during the experiment. Subsequently, participants will see that the confederate chose the same colour avatar as themselves. Half of the participants will read that their co-participant will perform the same tasks with the same paintings and words (same stimulus condition), and the other half will read that their co-participant will see different stimuli (different stimulus condition). To ensure that participants view stimuli in parallel, the experimenter will initiate stimulus presentation. During the recognition task, participants will wear headphones to minimise distractions from the confederate’s keypresses.

The remainder of the experiment will be the same in both conditions. Participants will perform a distractor task in which they will rate five paintings from the art pics database (Thieleking et al., 2020) on an 11-point Likert scale. Next, we will present participants with a random subset of 32 five-letter words from Aka et al. (2021) (e.g., board, razor, stone). These words will be matched^1^ on valence, arousal, and recall probability. These words will appear sequentially and in random order, for 1500 ms each, with a 250 ms fixation cross in between.

After a waiting screen, participants will begin the recognition task. The task will feature 64 words: the originally presented words plus 32 distractors fitting the same criteria. Each word will appear for 3000ms, or until the participant presses one of two keys to indicate whether they think the word is from the original list or a distractor. The key-decision mappings will be counterbalanced between participants. A 250 ms fixation will precede each word.

Following the experiment, participants will complete a short questionnaire indicating their age, gender, and any issues with their vision. The questionnaire will also feature two open-ended questions about participants’ experiences during the task. These will ask: (1) ‘What did you think this study was investigating?’, and (2) ‘Did you notice anything unusual or make any observations during the study?’. As an attention check, we will ask participants to identify the avatar colour they chose at the beginning of the task.

### Data analysis

We plan to exclude participants if (1) they report suspicion that their partner was a confederate, (2) they report being colour-blind, (3) their recognition accuracy is more than 2.5 standard deviations below the mean, and (4) they fail the post-experiment attention check. Two research assistants will independently rate questionnaire responses to determine whether we should exclude participants based on the first criterion^2^. We will recruit additional participants to replace those excluded to ensure adequate power.

For the recognition task, we will analyse d’ scores (i.e., the difference between z-scores for hit rates and false alarm rates). We will use a log-linear approach to deal with hit or false alarm rates of zero. This involves adding 0.5 to the number of hits and false alarms while adding 1 to the number of target and distractor trials before computing d’ (Stanislaw & Todorov, 1999). We will conduct a between-subjects *t*-test for the effect of group membership on recognition accuracy. If a Levene’s test reveals that variances are unequal between conditions, we will make inferences based on a Welch test instead.

As an additional measure of cognitive accessibility, we will likewise analyse response times (RTs) for hits, under the assumption that faster responses reflect greater accessibility. We will treat RTs faster than 200ms as anticipatory responses and exclude them from analysis. For all t-tests, we will also conduct equivalence tests using the two one-sided test (TOST) procedure (Lakens et al., 2018). The TOST procedure can provide evidence *for* the null hypothesis by testing whether the difference between two means is within two equivalence bounds. We will set the upper and lower equivalence bounds to *d* = 0.2 and − 0.2, respectively, as effects within this range can be considered trivial (Cohen, 1992). Data and analysis scripts will be available on osf.io.

## Experiment 2

Unless Experiment 1 finds no effect (indicated by the equivalence test) of co-attention, we will conduct a second experiment to examine whether such an effect occurs even when one’s partner is not an ingroup member. To this end, Experiment 2 will replicate Experiment 1 but without a group induction. Since related literature shows that shared attention augments our experience of stimuli even with nearby strangers (Boothby et al., 2014; Shteynberg et al., 2016), we expect the difference between the same vs. different stimuli conditions to persist.

## Method

### Participants

To ensure adequate power, we will conduct a power analysis based to detect an effect 10% smaller than found in Experiment 1. If necessary, we will add one participant to each condition to achieve an even number, enabling us to counterbalance key-response mappings.

### Procedure

The procedure will be identical to Experiment 1, but without an avatar selection process.

### Data analysis

Exclusion criteria and data analyses will be identical to Experiment 1, except there will be no attention check regarding avatar selection.

## Data Availability

No datasets were generated or analysed during the current study.

## Footnotes

1 We will match these properties by selecting only those words that fell within one standard deviation of the mean rating for each measure in Aka et al. (2021).

2 Conflicting ratings will be resolved via discussion.
